# Supplementary material for: Blood Culture Headspace Gas Analysis Enables Early Detection of Escherichia coli Bacteremia in an Animal Model of Sepsis
Source: Antibiotics (Basel). 2022 Jul 23;11(8):992. doi: 10.3390/antibiotics11080992 (PMC9331843; doi:10.3390/antibiotics11080992)
Supplement: Supplementary file 1 [file antibiotics-11-00992-s001.zip › Figure S1.pdf]

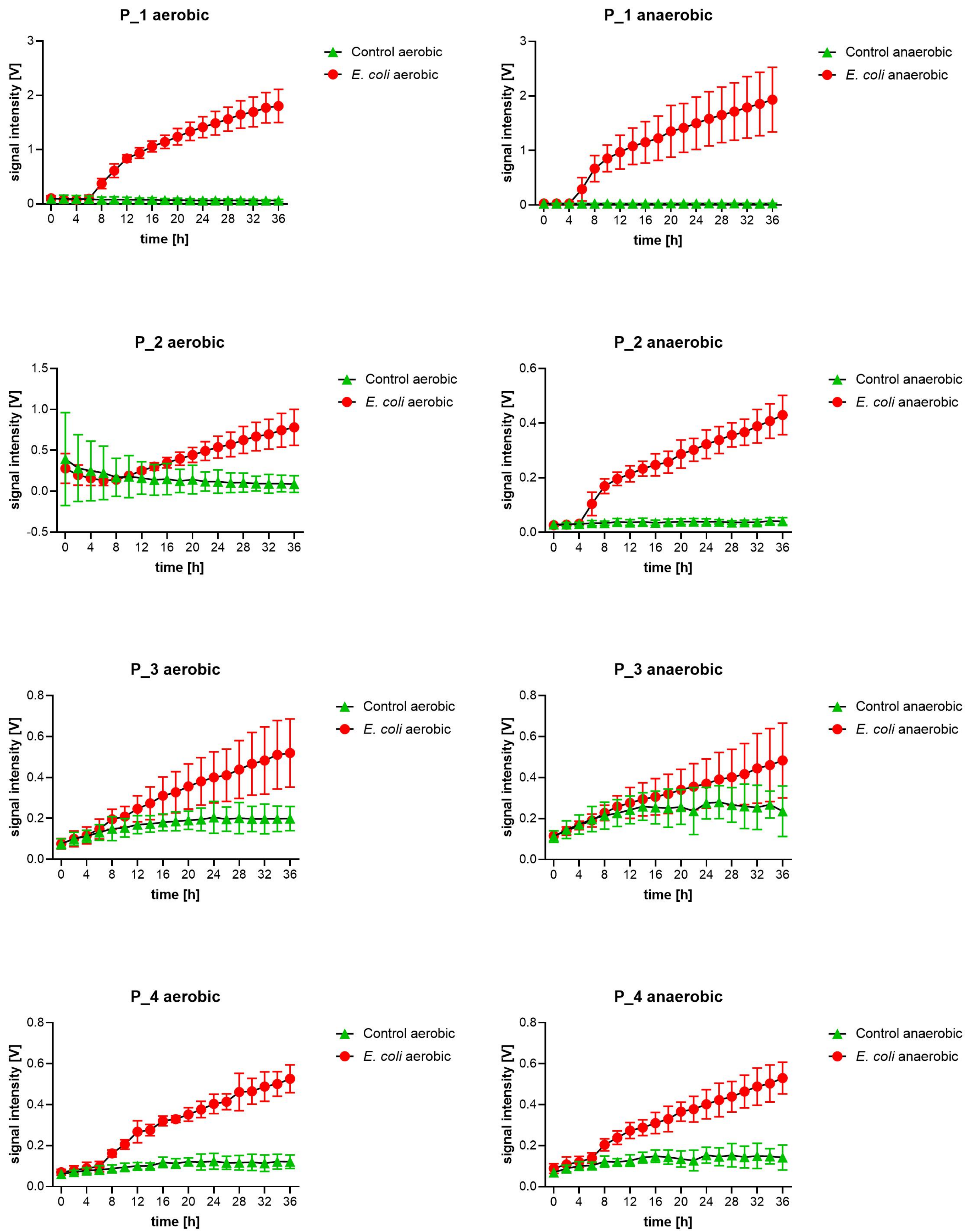
P_1 aerobic
Control aerobic
E. coli aerobic
P_1 anaerobic
Control anaerobic
E. coli anaerobic
P_2 aerobic
Control aerobic
E. coli aerobic
P_2 anaerobic
Control anaerobic
E. coli anaerobic
P_3 aerobic
Control aerobic
E. coli aerobic
P_3 anaerobic
Control anaerobic
E. coli anaerobic
P_4 aerobic
Control aerobic
E. coli aerobic
P_4 anaerobic
Control anaerobic
E. coli anaerobic
signal intensity [V]
time [h]

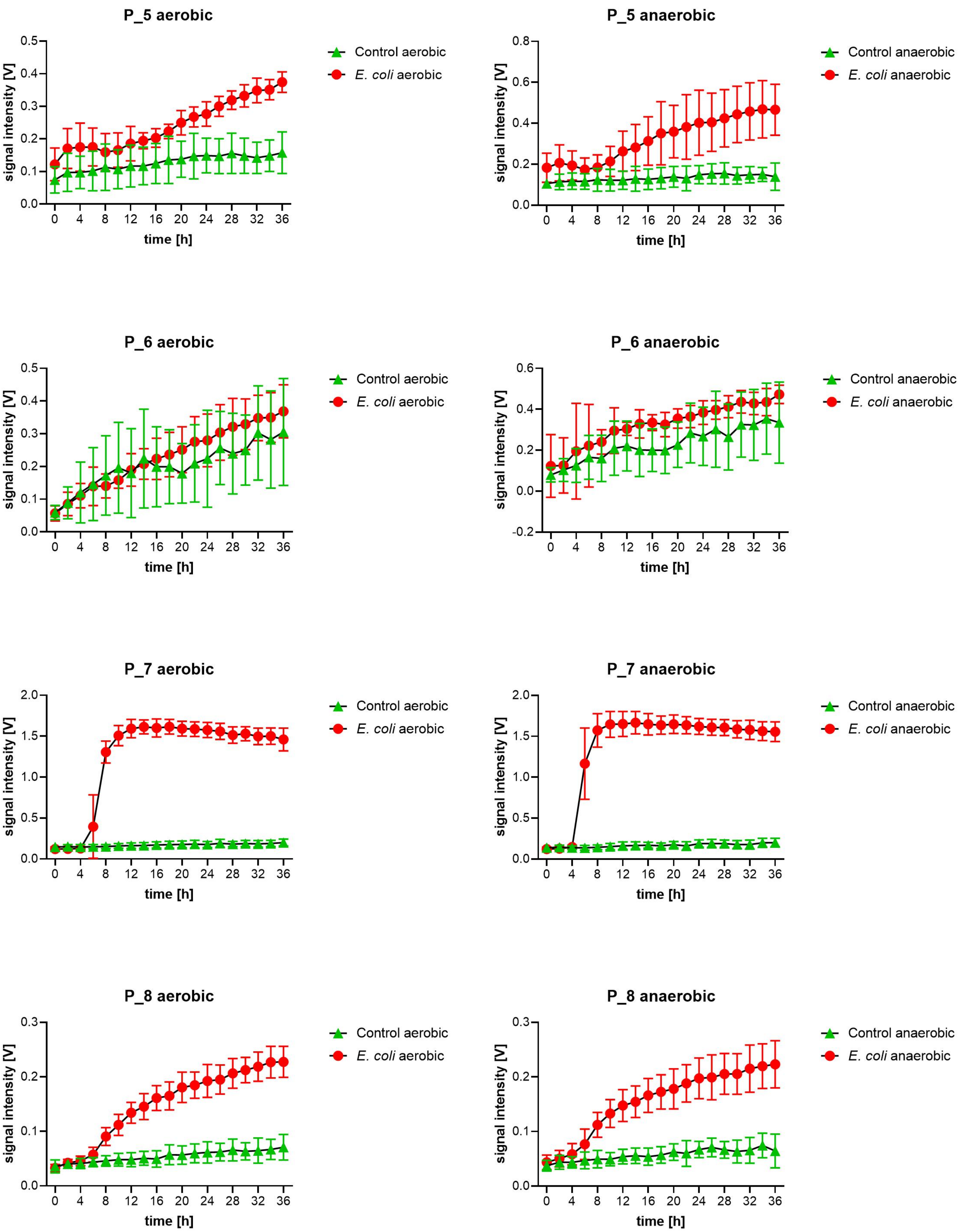
P_5 aerobic
P_5 anaerobic
P_6 aerobic
P_6 anaerobic
P_7 aerobic
P_7 anaerobic
P_8 aerobic
P_8 anaerobic
signal intensity [V]
time [h]
Control aerobic
E. coli aerobic
Control anaerobic
E. coli anaerobic

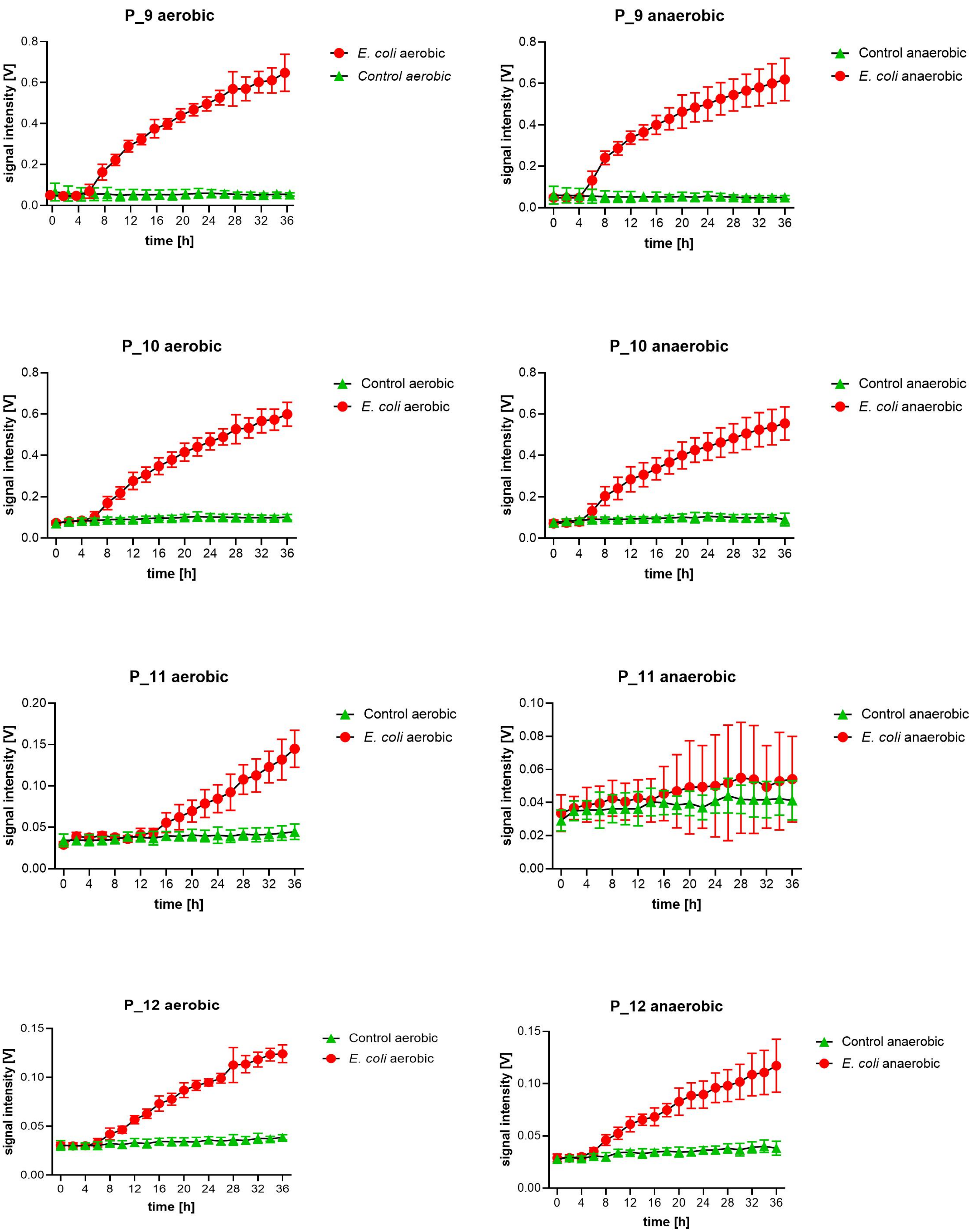
P_9 aerobic
E. coli aerobic
Control aerobic
P_9 anaerobic
Control anaerobic
E. coli anaerobic
P_10 aerobic
Control aerobic
E. coli aerobic
P_10 anaerobic
Control anaerobic
E. coli anaerobic
P_11 aerobic
Control aerobic
E. coli aerobic
P_11 anaerobic
Control anaerobic
E. coli anaerobic
P_12 aerobic
Control aerobic
E. coli aerobic
P_12 anaerobic
Control anaerobic
E. coli anaerobic
signal intensity [V]
time [h]
0 4 8 12 16 20 24 28 32 36

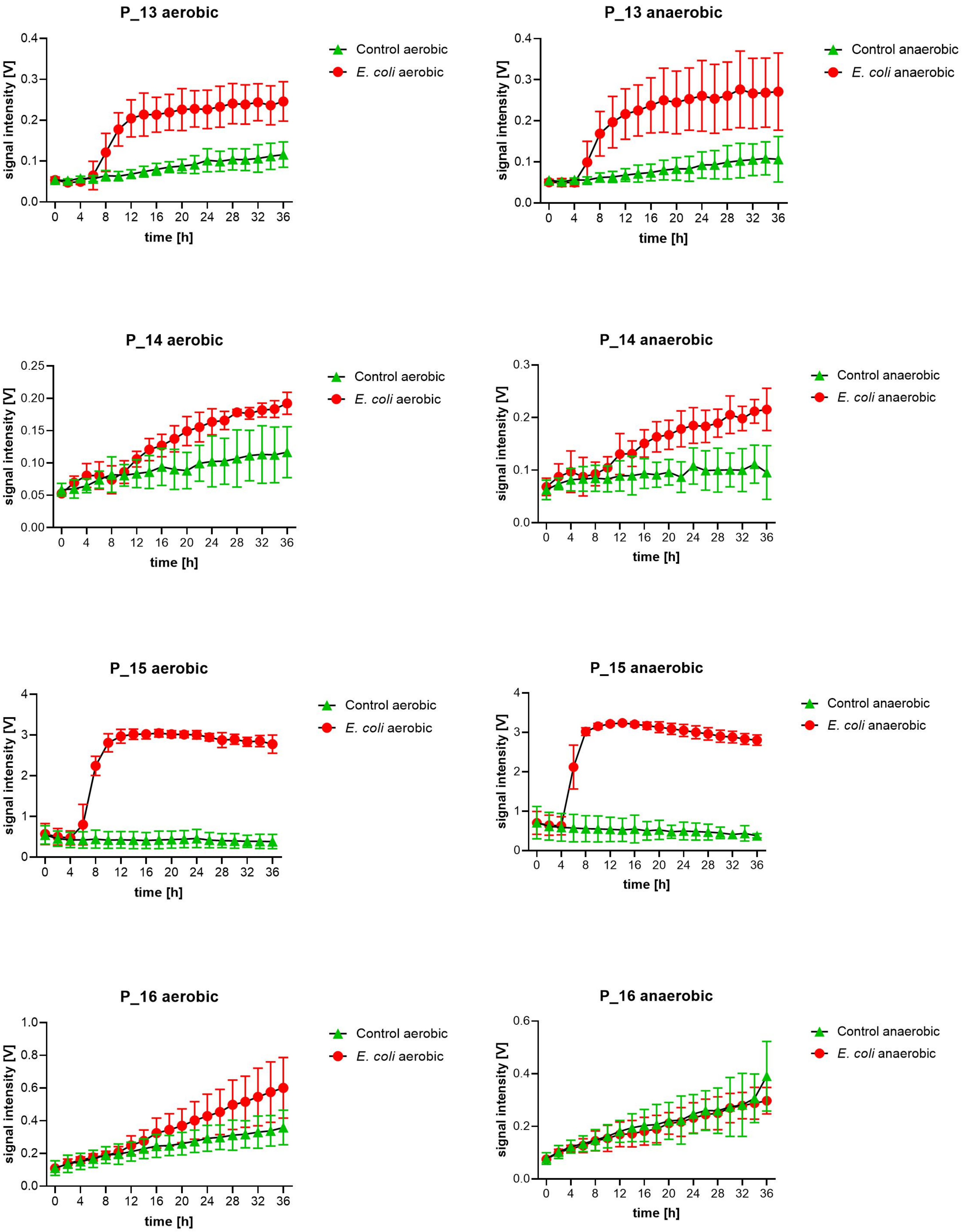
P_13 aerobic
signal intensity [V]
time [h]
Control aerobic
E. coli aerobic
P_13 anaerobic
signal intensity [V]
time [h]
Control anaerobic
E. coli anaerobic
P_14 aerobic
signal intensity [V]
time [h]
Control aerobic
E. coli aerobic
P_14 anaerobic
signal intensity [V]
time [h]
Control anaerobic
E. coli anaerobic
P_15 aerobic
signal intensity [V]
time [h]
Control aerobic
E. coli aerobic
P_15 anaerobic
signal intensity [V]
time [h]
Control anaerobic
E. coli anaerobic
P_16 aerobic
signal intensity [V]
time [h]
Control aerobic
E. coli aerobic
P_16 anaerobic
signal intensity [V]
time [h]
Control anaerobic
E. coli anaerobic

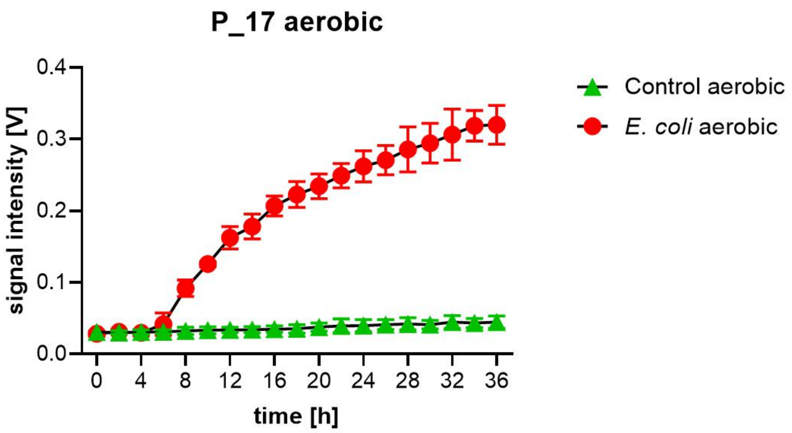
P_17 aerobic
signal intensity [V]
0.0
0.1
0.2
0.3
0.4
0 4 8 12 16 20 24 28 32 36
time [h]
Control aerobic
E. coli aerobic

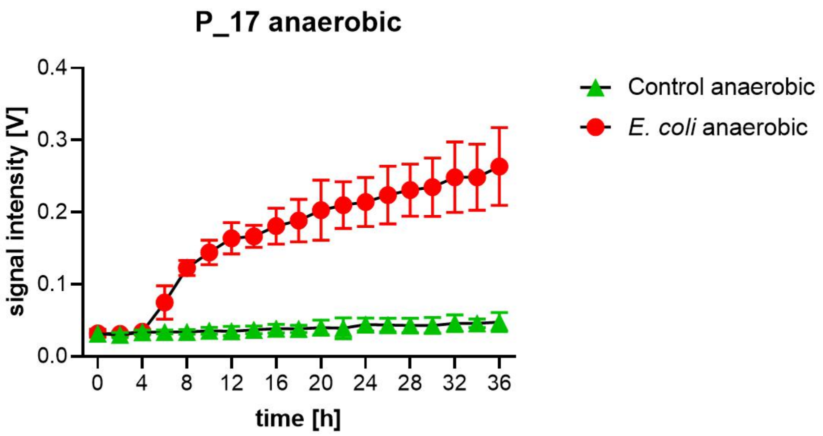
P_17 anaerobic
signal intensity [V]
0.0
0.1
0.2
0.3
0.4
0 4 8 12 16 20 24 28 32 36
time [h]
Control anaerobic
E. coli anaerobic

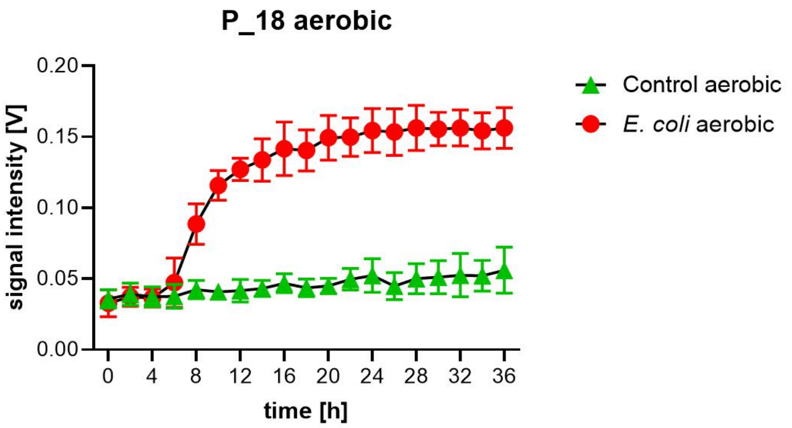
P_18 aerobic
signal intensity [V]
0.00
0.05
0.10
0.15
0.20
0 4 8 12 16 20 24 28 32 36
time [h]
Control aerobic
E. coli aerobic

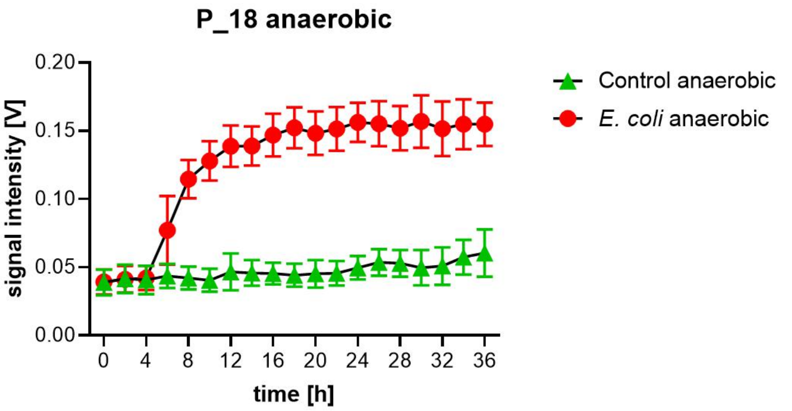
P_18 anaerobic
signal intensity [V]
0.00
0.05
0.10
0.15
0.20
0 4 8 12 16 20 24 28 32 36
time [h]
Control anaerobic
E. coli anaerobic

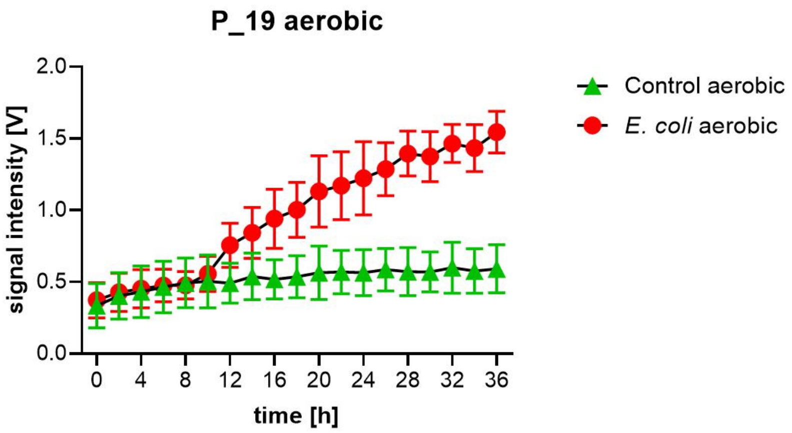
P_19 aerobic
signal intensity [V]
0.0
0.5
1.0
1.5
2.0
0 4 8 12 16 20 24 28 32 36
time [h]
Control aerobic
E. coli aerobic

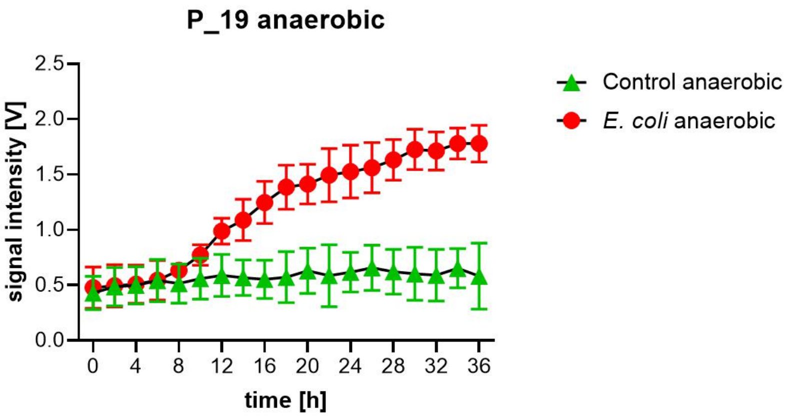
P_19 anaerobic
signal intensity [V]
0.0
0.5
1.0
1.5
2.0
2.5
0 4 8 12 16 20 24 28 32 36
time [h]
Control anaerobic
E. coli anaerobic

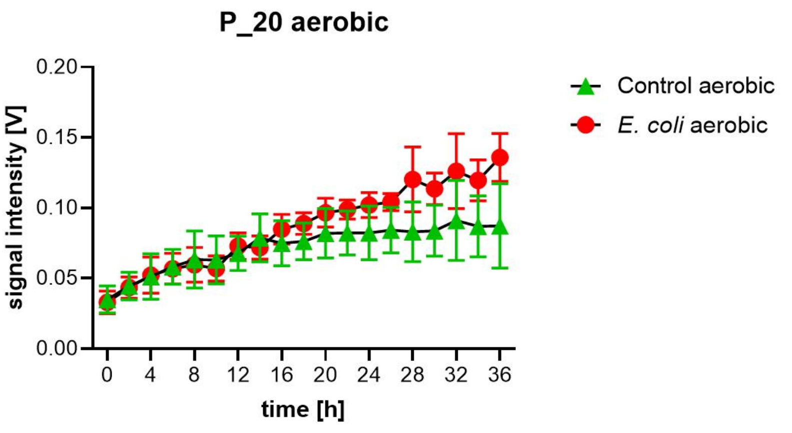
P_20 aerobic
signal intensity [V]
0.00
0.05
0.10
0.15
0.20
0 4 8 12 16 20 24 28 32 36
time [h]
Control aerobic
E. coli aerobic

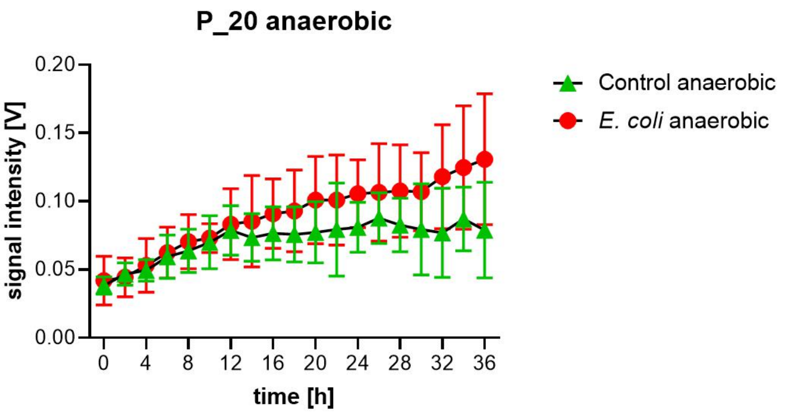
P_20 anaerobic
signal intensity [V]
0.00
0.05
0.10
0.15
0.20
0 4 8 12 16 20 24 28 32 36
time [h]
Control anaerobic
E. coli anaerobic

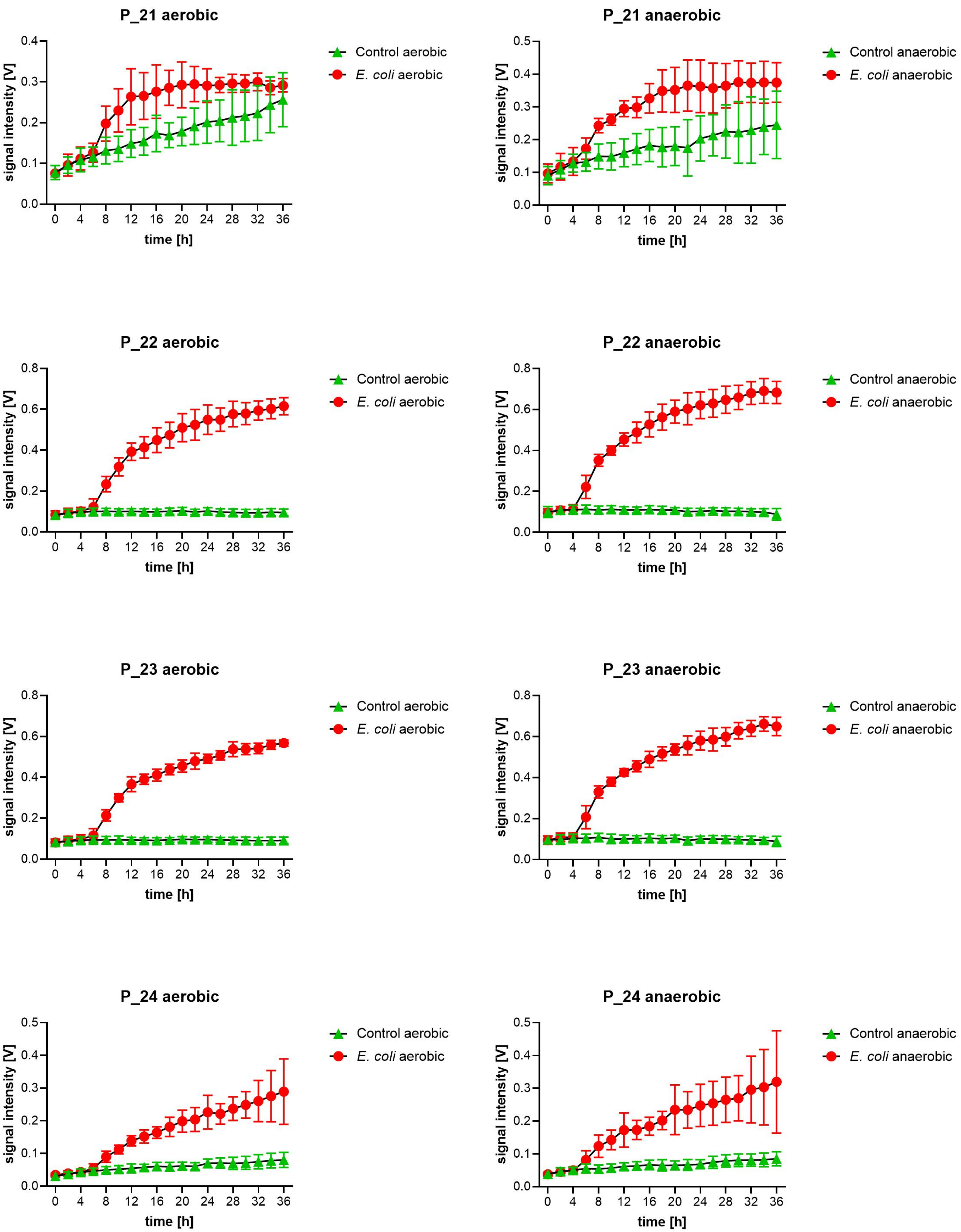
P_21 aerobic
P_21 anaerobic
P_22 aerobic
P_22 anaerobic
P_23 aerobic
P_23 anaerobic
P_24 aerobic
P_24 anaerobic
signal intensity [V]
time [h]
Control aerobic
E. coli aerobic
Control anaerobic
E. coli anaerobic
