# Supplementary figures and images for: Blood Culture Headspace Gas Analysis Enables Early Detection of Escherichia coli Bacteremia in an Animal Model of Sepsis
Source: Antibiotics (Basel). 2022 Jul 23;11(8):992. doi: 10.3390/antibiotics11080992 (PMC9331843; doi:10.3390/antibiotics11080992)

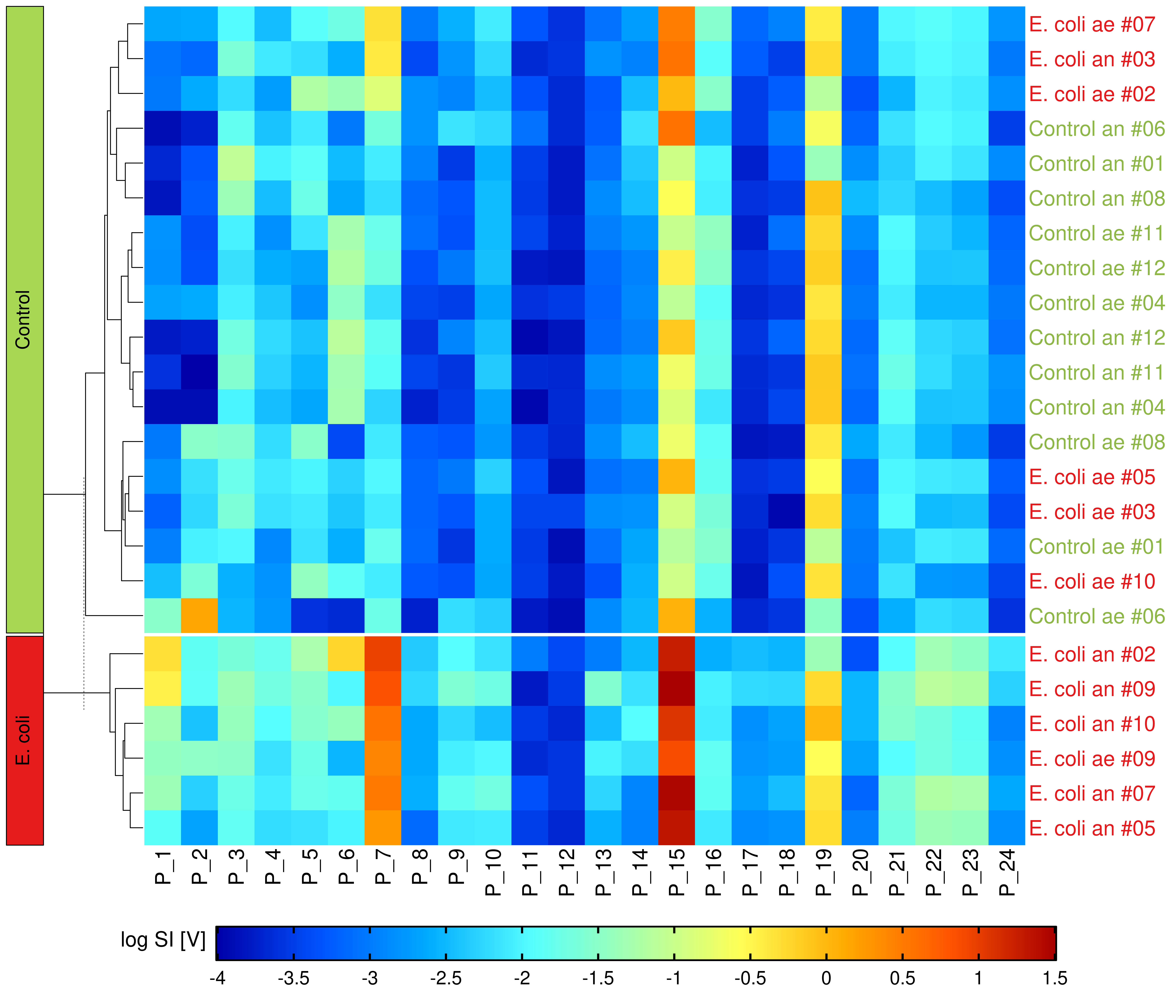

Supplement: Supplementary file 1 [file antibiotics-11-00992-s001.zip › Figure S2.png]
